# Inhibition of microRNA-451 is associated with increased expression of Macrophage Migration Inhibitory Factor and mitgation of the cardio-pulmonary phenotype in a murine model of Bronchopulmonary Dysplasia

**DOI:** 10.1186/s12931-020-01353-9

**Published:** 2020-04-22

**Authors:** Margaret Gilfillan, Pragnya Das, Dilip Shah, Mohammad Afaque Alam, Vineet Bhandari

**Affiliations:** 1 Department of Pediatrics, Drexel University College of Medicine, Philadelphia, PA 19103 USA; 2 St Christopher’s Hospital for Children, Philadelphia, PA 19134 USA; 3 Neonatology Research Laboratory, Education and Research Building, Cooper University Hospital, (Room #206), Camden, NJ 08103 USA; 4 Temple University, Philadelphia, PA 19140 USA; 5 Pediatrics, Obstetrics and Gynecology and Biomedical Sciences, Cooper Medical School of Rowan University, Camden, NJ 08103 USA; 6 Neonatology, The Children’s Regional Hospital at Cooper, One Cooper Plaza, Camden, NJ 08103 USA

**Keywords:** miR-451, Hyperoxia, Alveolarization, Lung, Angiopoietin, Vascular endothelial growth factor, Newborn

## Abstract

**Background:**

Macrophage migration inhibitory factor (MIF) has been implicated as a protective factor in the development of bronchopulmonary dysplasia (BPD) and is known to be regulated by MicroRNA-451 (miR-451). The aim of this study was to evaluate the role of miR-451 and the MIF signaling pathway in in vitro and in vivo models of BPD.

**Methods:**

Studies were conducted in mouse lung endothelial cells (MLECs) exposed to hyperoxia and in a newborn mouse model of hyperoxia-induced BPD. Lung and cardiac morphometry as well as vascular markers were evaluated.

**Results:**

Increased expression of miR-451 was noted in MLECs exposed to hyperoxia and in lungs of BPD mice. Administration of a miR-451 inhibitor to MLECs exposed to hyperoxia was associated with increased expression of MIF and decreased expression of angiopoietin (Ang) 2. Treatment with the miR-451 inhibitor was associated with improved lung morphometry indices, significant reduction in right ventricular hypertrophy, decreased mean arterial wall thickness and improvement in vascular density in BPD mice. Western blot analysis demonstrated preservation of MIF expression in BPD animals treated with a miR-451 inhibitor and increased expression of vascular endothelial growth factor-A (VEGF-A), Ang1, Ang2 and the Ang receptor, Tie2.

**Conclusion:**

We demonstrated that inhibition of miR-451 is associated with mitigation of the cardio-pulmonary phenotype, preservation of MIF expression and increased expression of several vascular growth factors.

## Background

Bronchopulmonary dysplasia (BPD) is a common and debilitating complication of prematurity that occurs primarily in infants who require supplemental oxygen and mechanical ventilation [1]. BPD affects up to 40% of infants born at less than 29 weeks gestational age [2] and is characterized by a phenotype of impaired lung development indicated by the presence of large immature alveoli [1, 3]. Dysregulated vascular growth also occurs in BPD and may lead to the development of pulmonary hypertension [4, 5]. Despite numerous advances in clinical care; the incidence of BPD has remained relatively static and the identification of new therapeutic targets continues to be a priority [6, 7].

Macrophage migration inhibitory factor (MIF) is a pluripotent cytokine that acts as a regulator of the innate immune system, encourages cell proliferation and activates pro-angiogenic pathways [8, 9]. When MIF was measured in the tracheal aspirates of preterm infants intubated for management of respiratory distress syndrome (RDS), infants with lower levels were found be more likely to develop BPD [10]. This association was demonstrated again in a larger study that also confirmed that a single nucleotide polymorphism that increases expression of MIF is independently associated with reduced risk for BPD [11]. When MIF levels were manipulated using MIF null mutant or knock out (MIFKO) and MIF overexpressing transgenic mice both extremes were associated with the development of an abnormal pulmonary phenotype [12, 13]. These studies strongly suggest a key role for MIF in normal lung development and injury and indicate the need to more closely characterize mechanisms that control MIF expression in both physiological and pathophysiological conditions.

MicroRNA-451 (miR-451) is a 22 base pair (bp) sequence of complementary nucleotides that has been shown to bind to the 3’untranslated (UTR) region of MIF mRNA leading to inhibition of protein synthesis [14, 15]. miR-451 has also been shown to reduce angiogenesis in colorectal cancer cells by targeting the interleukin – 6 receptor (IL-6R) [16] and to decrease T-cell responses to infection by inhibiting the expression of the regulatory gene, myc [17]. By inhibiting the expression of the regulatory protein Tyrosine 3-Monooxygenase/Tryptophan 5-Monooxygenase Activation Protein Zeta (YWHAZ), miR-451 has been found to promote pro-apoptotic pathways involving the transcription factor forkhead box O3 (FOXO3) [18, 19] and to reduce expression of pro-inflammatory cytokines in dendritic cells exposed to influenza virus [20].

Although miR-451 mediated inhibition of MIF and other proteins promoting angiogenesis and cell division has been described in malignant cell lines, these regulatory relationships have not yet been investigated within the context of BPD. Our goal was to study the effect of hyperoxia on miR-451 expression in both murine lung endothelial cells (MLECs) and in a previously validated murine model of hyperoxia-induced BPD. MLECs were chosen for our in-vitro model as we had a particular interest in the role of miR-451 as an anti-angiogenic regulator. After establishing that miR-451 does increase significantly in both MLECs exposed to hyperoxia and in the lung tissues of BPD mice, we proceeded to evaluate the effect of a miR-451 inhibitor on the cardio-pulmonary phenotype, expression of MIF, inflammatory markers and vascular growth factors in newborn (NB) mice exposed to room air (RA) and BPD conditions.

## Methods and materials

### Animals

All in vivo experiments were performed using wild type (WT) C57BL/6 mice purchased from the Jackson Laboratory (Bar Harbor, ME). Mice were housed at the Drexel University animal care facility. All animal experiments were approved by the Institutional Animal Care and Use Committee (IACUC) of Drexel University.

### Hyperoxia exposure

Hyperoxia exposure was performed using a method previously described [21, 22]. Briefly, NB mice were kept in an airtight Plexiglass container (55 × 40 × 50 cm) along with their mothers. A total of 32 pups, supported by 2 lactating dams were used to perform the experiments. Each litter comprising of 6 to 8 pups were randomized to either BPD or RA conditions. Animals in the BPD group were exposed to 100% oxygen from postnatal day (PN) 1 to PN4 which corresponds to the saccular stage of murine lung development. Mice were then allowed to recover in RA until PN14 when sacrifice was performed. Oxygen levels were measured continuously during the exposure period and the inside of the chamber was kept at atmospheric pressure. Lactating dams were cycled between the RA and hyperoxia groups every 24 h. Animals had free access to standard food and water and were subjected to a 12-h light-dark cycle. Survival was noted to be 100% which is consistent with previous work utilizing this experimental model of BPD [12, 23]. There was no significant difference in body weight between the groups at the end-point of the study (PN14).

### Cell culture

MLECs were purchased from Cell Biologics (Chicago, IL) and maintained in cell culture medium (Cell Biologics, Chicago, IL) as previously described [24]. Exposure to hyperoxia was achieved by leaving the plates inside a tightly sealed modular chamber (Stem Cell Technologies, Vancouver, Canada) filled with 100% oxygen for 16 h.

### In-vitro inhibition of miR-451

When MLECs had reached a confluence of approximately 70%, they were transferred to an antibiotic free growth medium (Cell Biologics, Chicago, IL) and transfected with 50 nM miR-451 inhibitor (catalog ID: IH-310630-07, Dharmacon, Lafayette, CO) using the Lipofectamine 3000 kit (Invitrogen, Thermo Fisher Scientific, Waltham, MA). Dosage of the inhibitor was determined based on the results of previous work published by our group [24, 25]. Cells were then re-plated and incubated at 37 °C for 24 h prior to exposure to either RA or hyperoxia.

### In-vivo inhibition of miR-451

A subgroup of mice in both the RA (*n* = 7) and BPD (*n* = 8) groups were treated intranasally with 20 μM of an oligonucleotide based miR-451 inhibitor (Qiagen, Valencia, CA) on PN2 and PN4. Dosage of the inhibitor was determined based on the results of previous work performed in our laboratory [23, 25].

### Bronchoalveolar lavage (BAL)

BAL specimens were obtained, and cell count analysis performed as previously described [21, 24]. Total protein content of BAL fluid was evaluated using the Pierce™ BSA Assay Kit (Thermo Fisher Scientific, Waltham, MA) as previously described [24, 26].

### Real-time reverse transcription PCR

RNA was extracted from both murine lung tissue and MLECs using the miRNeasy mini kit (Qiagen, Valencia, CA). Real-time reverse transcription PCR was performed as previously described [23]. miScript primer assay IDs MIMAT0001632 and MS00033740 were used for miR-451 and RNU6 respectively.

### Histological analysis

Lung tissues obtained from NB mice underwent a standardized inflation protocol (25 cm H_2_O) and were fixed in 4% paraformaldehyde. Specimens were then embedded in paraffin and 5 μm sections were obtained prior to staining with hematoxylin and eosin (H&E). This preparation was performed at the Department of Pathology Core Facility (Drexel University College of Medicine). Two random sections of both heart and lung tissues were obtained per animal with 6–8 animals represented in each experimental group.

### Lung morphometric analysis

All images for morphometric analysis were captured on an Olympus IX70 with DP73 camera attachment. At least 4 to 7 low power (magnification × 20) images were acquired for each animal with care taken to avoid capturing vessels and large airspaces. Alveolar size was estimated by measuring the mean chord length of the airspace automatically using ImageJ as previously described [23]. Four – 7 separate readouts were obtained for each animal. This software was also used to measure septal thickness. Measurements of radial alveolar counts were obtained as previously described [27].

### Measurement of BPD induced right ventricular hypertrophy (RVH)

Quantification of right ventricular (RV) wall thickness, left ventricular (LV) wall thickness and interventricular septal (IVS) thickness was performed by examining H&E stained specimens under 40x magnification using Cell Sens Olympus software. These measurements were then used to calculate the ratio of RV/(LV + IVS).

### Elastin staining

Elastin staining was performed on 5 μm thick lung paraffin sections using the Modified Verhoff’s elastin staining protocol of Percival and Radi [28]. The arterial thickness was measured by drawing an arbitrary line on the arterial wall using Olympus Cell Sens software (version 7).

### Immunostaining

Immunostaining was done following the methodology as previously described [23]. Briefly, 5um paraffin slides were dewaxed and dehydrated through a series of graded alcohol, followed by antigen retrieval in citrate buffer (pH 6) and incubation with vWF (DAKO, 1:100, Germany) at 4 °C, overnight. The following day, slides were washed 5 times with 1X PBS, 5 min each wash and incubated with the appropriate secondary antibody at room temperature for 2 h, washed again with 1X PBS for 5 times, 5 min each wash and mounted with vectashield 4′,6-diamidino-2-phenylindole (DAPI) (Vector laboratories, CA).

For imaging, the slides were manually counted under high power field for the number of vessels to cover the entire lung. Five – 6 animals were taken for each group. Photomicrographs were taken at 10X and 40X magnifications, and intensity adjusted with Adobe Photoshop 13.

### Western blotting

Detection of MIF, angiopoietin (Ang)1, Ang 2, tyrosine kinase with immunoglobulin and epidermal growth factor homology domains receptor 2 (Tie2), Vascular Endothelial Growth Factor-A (VEGF-A), interleukin (IL)-6, IL-1β, YWHAZ and FOXO3 was performed using vinculin and β-actin as loading controls from lung tissue and MLEC lysates using Western blot as previously described [25].

The primary antibodies used were MIF (Abcam, Cambridge, UK, 1:400), Vinculin (Santa-Cruz Biotechnology, Dallas, TX; 1:10,000), β-actin (Cell Signaling Technology, Danvers, MA), Ang1, (Sigma-Aldrich, St. Louis, MO, 1:500), Ang2 (Sigma-Aldrich, St. Louis, MO, 1:500), Tie2 (Santa-Cruz Biotechnology, Dallas, TX; 1:200), VEGF-A (Abcam, Cambridge, UK, 1:200), IL-6 (Santa-Cruz Biotechnology, Dallas, TX; 1:500), IL-1β (Cell Signaling Technology, Danvers, MA), FOXO3 (Cell Signaling Technology, Danvers, MA; 1:1000), YWHAZ, (Santa-Cruz Biotechnology, Dallas, TX; 1:800).

ImageJ software was used to analyze expression of target proteins relative to either vinculin or β-actin loading controls.

### Statistical analysis

Previous work has shown that mean chord length, as a measure of alveolarization is abnormal in > 99% of WT BPD mice lungs. We expected that treatment with a miR-451 inhibitor would yield an 80% improvement in mean chord length. Hence, with an alpha = 0.005, beta = 0.2, and a power of 80%, we calculated a need for *n* = 6 in each group. All statistical analysis was performed using Graph Pad Prism Version 7 (GraphPad software, San Diego, CA). Values are expressed as mean +/− SEM. Groups were compared using the Student’s two-tailed unpaired T-test or one-way analysis of variance (ANOVA) with Tukey’s post-hoc test to correct for multiple comparisons, where appropriate. A *p* value of < 0.05 was considered statistically significant.

## Results

### Expression of miR-451 is upregulated by hyperoxia in MLECs and in murine lung tissues

**Fig. 1:**
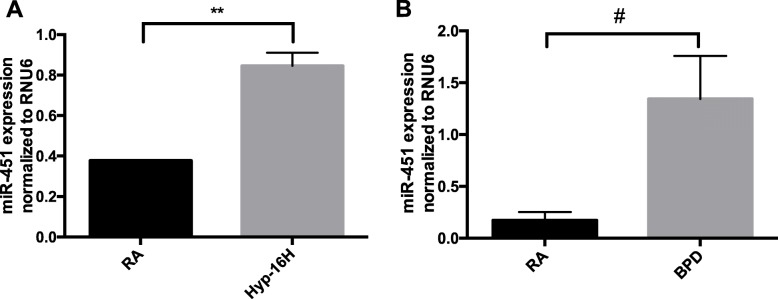
Expression of miR-451 is upregulated by hyperoxia in MLECs and in murine lung tissues. **a** RNA was extracted from MLECs grown in room air and exposed to hyperoxia (95% O_2_) for 16 h. miR-451 expression was evaluated using RT-qPCR. *N* = 3, in each group. **b** RT-qPCR was performed on RNA extracted from the lungs of NB room air (RA) and BPD mice. *N* = 5, in each group. MLECs: mouse lung endothelial cells, RA: room air, Hyp – 16 h: hyperoxia for 16 h; NB: newborn; RA: room air; BPD: bronchopulmonary dysplasia. ** *p* < 0.01 # *p* < 0.001. Data are expressed as mean ± SEM

In repeated experiments, miR-451 expression was noted to be significantly increased in both MLEC’s exposed to 100% O_2_ for 16 h (Fig. 1a) and in lung tissues of NB BPD mice (Fig. 1b). These data suggest that exposure to hyperoxia during the critical saccular phase of lung development leads to upregulation of miR-451 in endothelial cells. Similar findings were noted in fetal MLECs exposed to 60% O_2_ for 16 h (Supplemental Fig. 1). A trend (*p* = 0.13) towards increased expression of miR-451 was also noted in mouse lung epithelial cells (MLE12) cells exposed to hyperoxia for 16 h (Supplemental Fig. 2). It is therefore possible that upregulation of miR-451 may contribute to the decrease in MIF expression previously reported in this specific murine model of BPD [12].

### Inhibition of miR-451 is associated with increased expression of MIF in MLECs exposed to hyperoxia

**Fig. 2:**
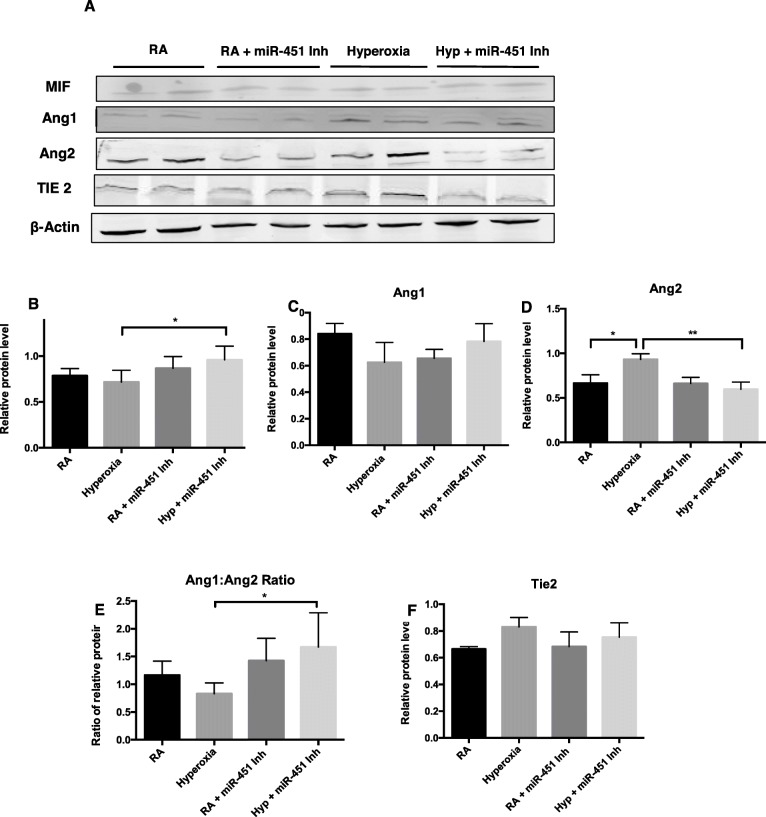
Inhibition of miR-451 is associated with increased expression of MIF in MLECs exposed to hyperoxia. **a** Representative image of Western blot analysis showing expression of macrophage migration inhibitory factor (MIF), Angiopoietin 1 (Ang 1), Ang 2, and their receptor, Tie 2 and β-actin in MLECs exposed to hyperoxia and transfected with a miR-451 inhibitor. **b** MIF expression quantified by densitometry with normalization to β-actin. **c** and **d** Expression of the vascular growth factors Ang1, Ang2 quantified by densitometry with normalization to β-actin. **e** Ratio of the densitometric values of Ang1 and Ang2. **f** Expression of the Ang receptor Tie2 quantified by densitometry with normalization to β-actin. N = 3–4, in each group. RA: room air, Hyp: hyperoxia 95% O_2_ for 16 h, miR-451 Inh: miR-451 inhibitor. * *p <* 0.05 **P < 0.01

After noting the significant increase in the expression of miR-451, a negative regulator of MIF, we questioned whether introducing a miR-451 inhibitor would have an effect on the expression of MIF and other angiogenic proteins in the MIF signaling pathway. Expression and quantification of MIF is shown in Fig. 2a and b. MIF expression in MLECs exposed to hyperoxia was not significantly different compared to RA controls; however, expression of MIF was significantly increased in the presence of hyperoxia and a miR-451 inhibitor. No significant changes were noted in the expression of Ang1 either in the presence of hyperoxia or with treatment with the miR-451 inhibitor (Fig. 2a and c**)**. Exposure to hyperoxia was associated with a significant increase in Ang2 expression in MLECs which is consistent with previous reported findings [29]. Transfection with a miR-451 inhibitor was associated with a significant decrease in Ang2 expression in the presence of hyperoxia compared to the hyperoxia control (Fig. 2a and d**)**. Changes in Ang2 expression resulted in a significant increase in the Ang1 to Ang2 ratio in MLECs exposed to both the miR-451 inhibitor and hyperoxia (Figs. 2e).

Ang1 and Ang2 compete for sites at the Tie2 receptor and changes in Tie2 expression have been previously noted in the presence of hyperoxia in lung tissue samples. Our results do not show any significant differences in Tie2 expression between the 4 experimental groups of MLECs (Fig. 2a and f**)**.

Overall, these findings demonstrate that inhibition of miR-451 in MLECs exposed to hyperoxia is associated with both increased expression of MIF and an increased Ang1/Ang2 ratio. These changes could potentially allow for increased capacity for healthy vascular growth when miR-451 activity is reduced. Although the maintenance of MIF expression in the hyperoxia control group was unexpected, there may be other factors counteracting the activity of miR-451 that we are not able to measure. It is also likely that the decrease in MIF expression noted in murine BPD lung tissues noted by Sun et al. [12] occurs due to interactions between different cell types.

### Inhibition of miR-451 is associated with increased neutrophils and protein in BAL fluid

**Fig. 3:**
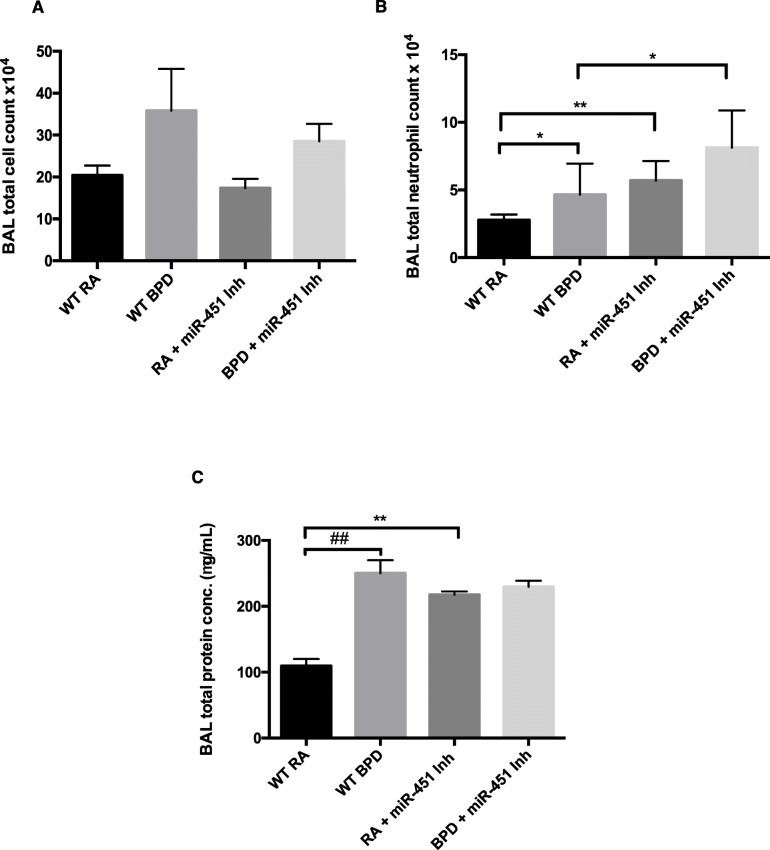
Inhibition of miR-451 is associated with increased neutrophils and protein in BAL fluid. **a** Total cell count values were obtained in RA and BPD mice, with and without treatment with a miR-451 inhibitor. Each bar represents the mean ± SEM of a minimum of 6 different animals. **b** BAL total neutrophil counts for WT RA and BPD mice, compared with those treated with a miR-451 inhibitor. Each bar represents the mean ± SEM of a minimum of 5 different animals. **c** BAL specimens from WT and miR-451 treated mice were evaluated for total protein content. Each bar represents the mean ± SEM obtained from a minimum of 7 different animals. BAL: bronchoalveolar lavage, RA: room air, BPD: bronchopulmonary dysplasia, WT: wild type, miR-451 Inh: miR-451 inhibitor treated. * *p* < 0.05, ** *p* < 0.01, #**#***p* < 0.0001

We proceeded to investigate the effect of a miR-451 inhibitor in a murine model of hyperoxia-induced BPD. Figure 3 shows the results of BAL fluid analysis, specifically BAL total cell count, BAL neutrophil count and BAL total protein values. We did not notice any significant differences in total cell counts between the four experimental groups, though trends were noted in the BPD groups compared to their respective RA controls (*p* = 0.081 for WT RA and WT BPD, *p* = 0.054 for miR-451 inhibitor treated RA and BPD groups). (Fig. 3a). Total neutrophil count was found to be significantly increased in the WT BPD BAL specimens compared to the WT RA specimens (Fig. 3b), a finding that is consistent with previous reports in the literature [12]. Total neutrophil counts were also found to be significantly increased in the miR-451 inhibitor treated RA group and elevated in the miR-451 inhibitor treated BPD group compared to the WT RA group and the WT BPD groups respectively. In WT BPD animals, BAL total protein was significantly increased (Fig. 3c). This finding has been previously reported in this specific murine model of hyperoxia induced BPD [25]. BAL total protein values were also found to be significantly elevated in the miR-451 inhibitor treated RA group compared to the WT RA control without any difference noted between the WT BPD group and the miR-451 inhibitor treated group (Fig. 3c).

Taken together, these findings are suggestive of increased inflammation occurring in the lungs of animals treated with a miR-451 inhibitor compared to WT animals. Interestingly, the increase in neutrophil count and total protein content in the miR-451 inhibitor treated groups is present in RA. Unlike in the control animals, the increase in total protein content does not appear to be potentiated by exposure to oxygen, suggesting that miR-451 may play a role in regulating proteins that are important in lung development.

### Effect of an miR-451 inhibitor on lung morphometry indices and pulmonary arterial hypertension (PAH) induced RVH

**Fig. 4:**
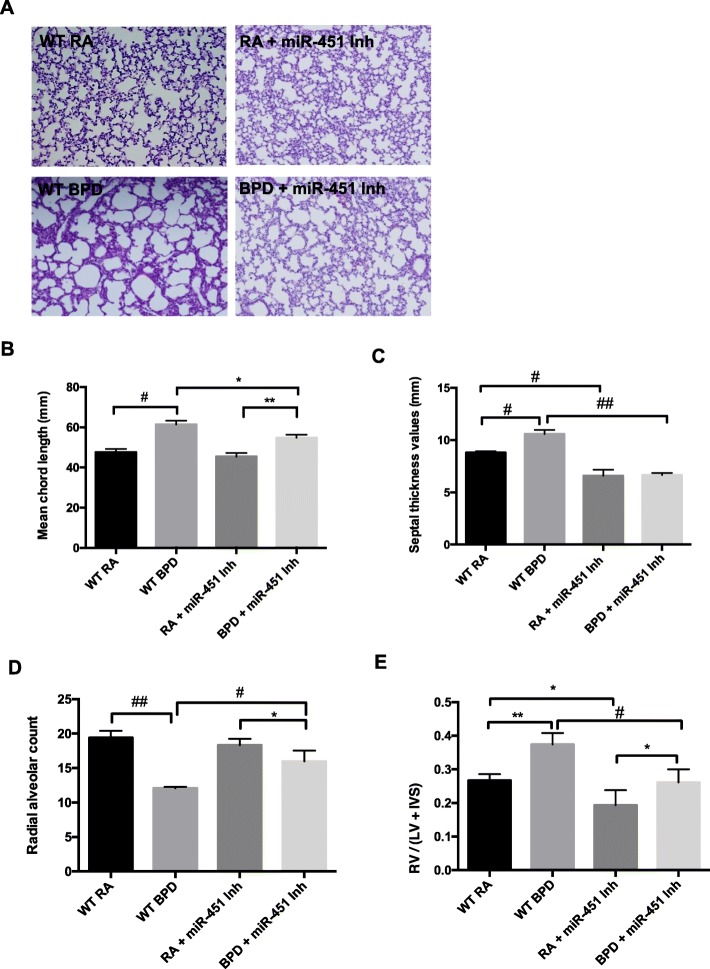
Effect of an miR-451 inhibitor on lung morphometry indices and pulmonary arterial hypertension (PAH)-induced right ventricular hypertrophy (RVH). **a** Representative photomicrographs of murine lung tissue prepared with H&E stain obtained from NB WT RA, WT BPD, miR-451 inhibitor treated animals. **b** Alveolar size evaluated by mean chord length, septal thickness and radial alveolar counts are shown. Each column is representative of the mean ± SEM of measurements obtained from a minimum of 5 animals. The ratio of right ventricular wall thickness to the combined measurement of the left ventricular and interventricular septal wall thickness (RV/(LV + IVS)) is also shown as an indicator of PAH-induced RVH. Each column is representative of the mean ± SEM of measurements obtained from a minimum of 4 animals. NB: newborn, WT: wild type, RA: room air, BPD: bronchopulmonary dysplasia. * *p* < 0.05, ** *p* < 0.01*,* # *p* < 0.001*,* ## *p* < 0.0001

The in-vivo model that we chose to study involves exposure of NB mice to 100% oxygen during the saccular stage of lung development followed by recovery a recovery period in RA*.* By introducing a miR-451 inhibitor during the middle and end of the 4-day period of oxygen exposure we hoped to look at the effect of altering miR-451 expression during both the window of lung injury and at the beginning of the 10-day recovery phase. Representative images shown in Fig. 4a demonstrate WT BPD lungs with large, oversimplified alveoli separated by thick septal walls. Treatment with a miR-451 inhibitor resulted in a significant improvement in alveolarization in BPD animals as evidenced by chord length (Fig. 4b) when compared to the WT BPD lungs. Septal thickness was also significantly decreased in the miR-451 inhibitor treated BPD group compared to the WT BPD group (Fig. 4c) and treatment with the miR-451 inhibitor was also associated with a significantly increased radial alveolar count in BPD exposed animals (Fig. 4d).

Measurements of RV wall thickness, LV wall thickness and IVS thickness were also obtained from animals in the four different experimental groups. The ratio of the RV to the combined measurement of the LV and IVS was used as an indicator of the degree of RV remodeling i.e. RVH secondary to PAH. As expected, in the WT BPD animals there was a significant increase in RVH. RVH was significantly reduced in both the inhibitor treated RA and BPD animals compared to their WT counterparts (Fig. 4e).

### Effect of a miR-451 inhibitor on vascular density and pathology

**Fig. 5:**
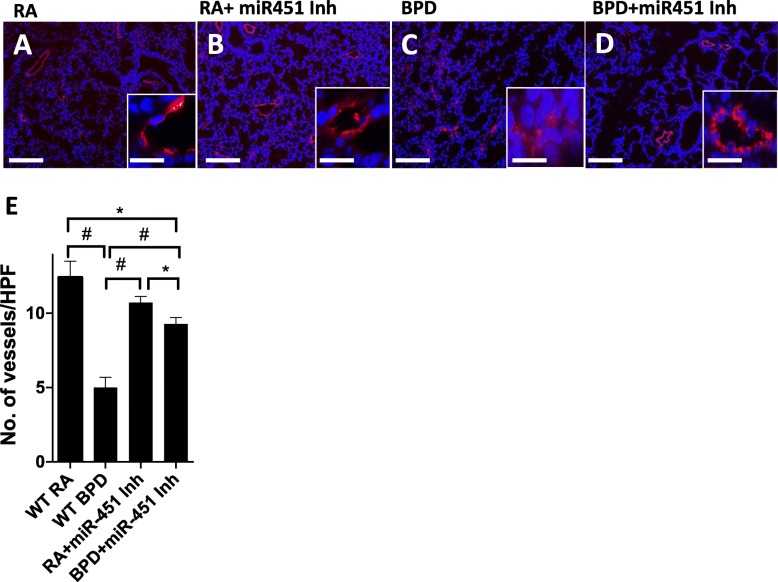
Effect of a miR-451 inhibitor on vascular vessel density measured by von Willebrand Factor (vWF) immunostaining. Immunostaining showing vascular staining of pulmonary vessels by vWF, a marker for blood vessels. In room air (RA), there are more vessels with intact vascular wall (**a**) as compared to the BPD group (**c**), where the vessels are disrupted and do not have smooth walls (see inset). After administration of miR-451 inh (**d**), the pulmonary phenotype of the alveolar sacs are restored, there are increased number of intact blood vessels, and the vascular wall regain their shape (inset). The inhibitor did not have any deleterious effect in the RA-treated group (**b**). Side panel shows quantification of blood vessels in different groups after counting each region of the lung in high power field. HPF: High power field; * *p* < 0.05; # *p* < 0.001; Scale Bar: 100 μm; inset scale bar: 10 μm. Blue: 4′,6-diamidino-2-phenylindole (DAPI); Red: vWF

In order to evaluate the effect of miR-451 inhibition on angiogenesis, we performed immunostaining for von Willebrand factor (vWF); a protein that is known to be a marker for blood vessels. A normal pattern of vascular density seen in the RA control group is demonstrated in Fig. 5a. This picture is similar to that noted in the miR-451 inhibitor treated RA group (Fig. 5b). In contrast, exposure to hyperoxia was noted to be associated with reduced vascular density (Fig. 5c) that appears to be mitigated by treatment with a miR-451 inhibitor (Fig. 5d). Manual counting of vWF stained blood vessels in each high-powered field (HPF) revealed significant attenuation of vascular density in the WT BPD mice compared to both the WT RA group and the miR-451 inhibitor treated BPD group (Fig. 5e). Although vascular density in the miR-451 inhibitor group remained significantly lower than in the WT RA and miR-451 inhibitor treated RA groups, it appears that inhibition of miR-451 does have some benefit in preserving angiogenesis in NB mice exposed to hyperoxia.

**Fig. 6:**
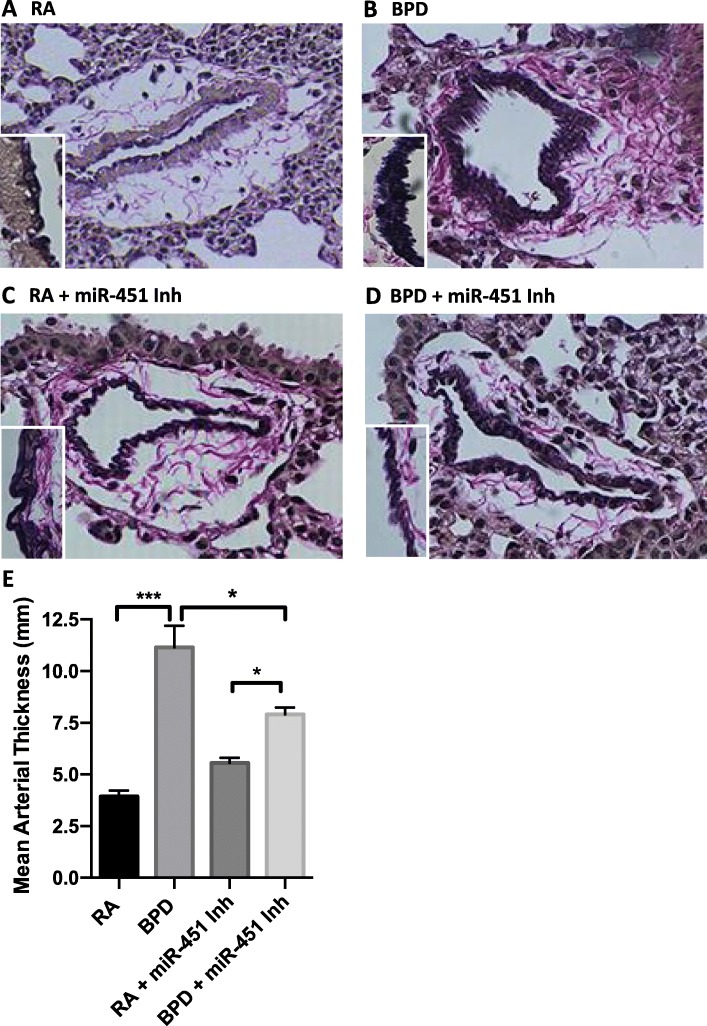
Effect of a miR-451 inhibitor on vascular remodeling shown using elastin staining and quantification of arterial wall thickness. Representative images of tissues obtained from NB WT RA (**a**), WT BPD (**b**), miR-451 inhibitor treated RA (**c**) and BPD animals (**d**) prepared with Verhoeff’s stain are shown to demonstrate changes in elastin deposition. Inset: vessel wall in high magnification (X400). Quantification of the mean arterial wall thickness in the four experimental groups is shown in (**e**). Each column is representative of data obtained from a minimum of 4 animals. * *p* < 0.05; ** p < 0. 01, *** p < 0.001 Scale Bar: 100 μm

Arterial wall pathology was assessed by elastin staining and the mean arterial thickness measured. Elastin present in blood vessels is arranged as dark concentrated bands, as is evident from the representative images of elastin staining shown in in Fig. 6a-d. In the RA group (Fig. 6a), the arterial wall showed a uniform deposition of elastin, while in the BPD group (Fig. 6b), the wall is thick, intense with an uneven wall. These changes are accompanied by heavy deposition of collagen around the vessel. Treatment with a miR-451 inhibitor was associated with overall improvement in the degree of vascular remodeling (Fig. 6d). Although the vessel walls still appear uneven and serrated, they are thinner in comparison to the BPD group, with less collagen deposition surrounding the vessel wall. Administration of the miR-451 inhibitor to mice studied under normoxic conditions (Fig. 6c) did not have much effect on the wall composition and density, but the collagen fibers were more prominent when compared to the RA control group. The significant decrease in mean arterial wall thickness in BPD mice who were treated with a miR-451 inhibitor is shown in Fig. 6e. These findings suggest that inhibition of miR-451 under hyperoxic conditions is associated with mitigation of vascular remodeling that could lead to pulmonary hypertension.

### Effect of a miR-451 inhibitor on the expression of MIF, vascular growth factors and inflammatory cytokines

**Fig. 7:**
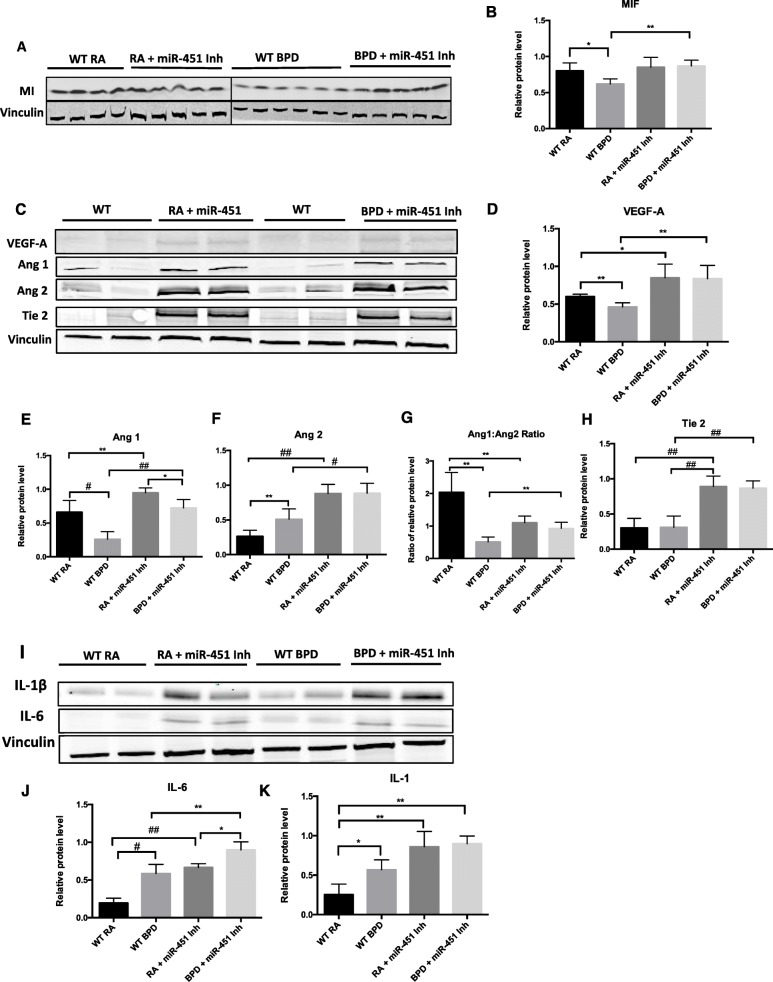
Effect of a miR-451 inhibitor on the expression of MIF, vascular growth factors, IL-1β and IL-6. **a** Representative western blot showing the expression of MIF. **b** MIF expression quantified by densitometry with normalization to vinculin. Each column of the graph represents the mean ± SEM of specimens obtained from a minimum of 4 different animals. **c** Representative western blot showing expression of Ang1, Ang2, Tie 2, VEGF-A and vinculin. **d-f** VEGF-A, Ang1 and Ang2 expression quantified by densitometry with normalization to vinculin. Each column is representative of the mean ± SEM of values obtained from a minimum of 4 different animals. **g** Ratio of the normalized expression of Ang1 to Ang2 with each column representative of the mean ± SEM of matched values obtained from a minimum of 4 different animals. **h** Tie2 expression quantified by densitometry with normalization to vinculin, each column represents the mean ± SEM of a minimum of 4 different animals. **i** Representative western blot showing expression of interleukin (IL)-6, IL-1β and vinculin. **j** and **k** IL-6 and IL-1β expression quantified by densitometry with normalization to vinculin. Columns are representative of the mean ± SEM of values obtained from a minimum of 3 different animals. * *p* < 0.05, ** *p* < 0.01*,* # *p* < 0.001*,* ## *p* < 0.0001

After noting that administration of a miR-451 inhibitor had a favorable effect on the pulmonary phenotype in this murine model of BPD, we then proceeded to evaluate the mechanism underlying these effects vis-a-vis the MIF signaling pathway. Figure 7a and b show that MIF expression was found to be robust in WT RA mice but significantly decreased in WT BPD mice, consistent with previous findings [12]. When a miR-451 inhibitor was administered, MIF expression in the BPD group was found to be comparable with levels found in mice in RA but significantly increased in comparison to the WT BPD group (Fig. 7a and b).

We went on to study the effect of introducing a miR-451 inhibitor on the expression of vascular growth factors regulated by MIF (Fig. 7c). VEGF-A expression was found to decrease significantly in response to hyperoxia in WT animals (Fig. 7c and d). When a miR-451 inhibitor was introduced, VEGF-A levels were found to be significantly elevated in BPD mice lungs in comparison to their WT counterparts. In miR-451 inhibitor treated mice, there was no difference in VEGF-A expression between the RA and the BPD group. Ang1 expression was significantly decreased in BPD mice (Fig. 7c and d). Expression of Ang1 was noted to be significantly higher in both the miR-451 inhibitor RA and BPD groups compared to their WT counterparts (Fig. 7c and e). The effect of hyperoxia exposure on Ang1 expression persisted even in the presence of the miR-451 inhibitor; however, Ang1 expression in miR-451 inhibitor treated BPD mice was significantly higher compared to WT BPD mice and not statistically different to Ang1 expression in the WT RA group (Fig. 7c and e).

Ang2 expression was noted to be significantly increased in WT BPD mice in comparison to the RA control group (Fig. 7c and f). Treatment with a miR-451 inhibitor was found to be associated with a marked increase in Ang2 expression that was noticeable to a similar degree in both RA and BPD mice. In contrast to the WT controls, where exposure to BPD conditions resulted in a significant increase in Ang2 expression, we did not detect any further increase in Ang2 levels following exposure of the miR-451 inhibitor treated animals to hyperoxia. This finding suggests that treatment with a miR-451 inhibitor was sufficient to provoke a maximal increase in Ang2 levels in the absence of hyperoxia exposure.

Treatment with a miR-451 inhibitor was associated with decreased Ang1:Ang2 ratio relative to that seen in the WT RA group in both RA and BPD mice; however, the values noted in the miR-451 inhibitor treated RA and BPD groups were significantly higher than that those noted in the WT BPD group (Fig. 7g). Interestingly, exposure to the BPD model was not associated with a significant decrease in Ang1:Ang2 ratio in mice treated with a miR-451 inhibitor. Exposure to a miR-451 inhibitor was also associated with a marked increase in Tie2 expression that was noted at in RA and persisted following exposure to the BPD model (Fig. 7c and h).

Expression of both IL-6 and IL-1β was found to be significantly elevated in WT BPD mice compared to the RA controls (Fig. 7i and j). In miR-451 treated animals the levels of both cytokines were markedly elevated both in RA and BPD animals in comparison to their WT counterparts. In the miR-451 inhibitor treated mice, IL-6 expression increased significantly following exposure to hyperoxia. No significant change in IL-1β expression was noted in BPD mice lungs with or without miR-451 inhibitor administration (Fig. 7i and k).

Taken together these findings suggest that miR-451 mediated inhibition of MIF expression may contribute to the decrease in MIF levels noted when NB mice are exposed to hyperoxia during the saccular phase of lung development. Changes in the expression of vascular growth factors downstream of MIF such as VEGF-A, Ang1, Ang2 and the Tie2 receptor could potentially be affected by miR-451 mediated regulation of MIF. Treatment with a miR-451 inhibitor is also associated with increased expression of the pro-inflammatory cytokines IL-6 and IL-1β.

### Effect of a miR-451 inhibitor on the expression of YWHAZ and FOXO3

**Fig. 8:**
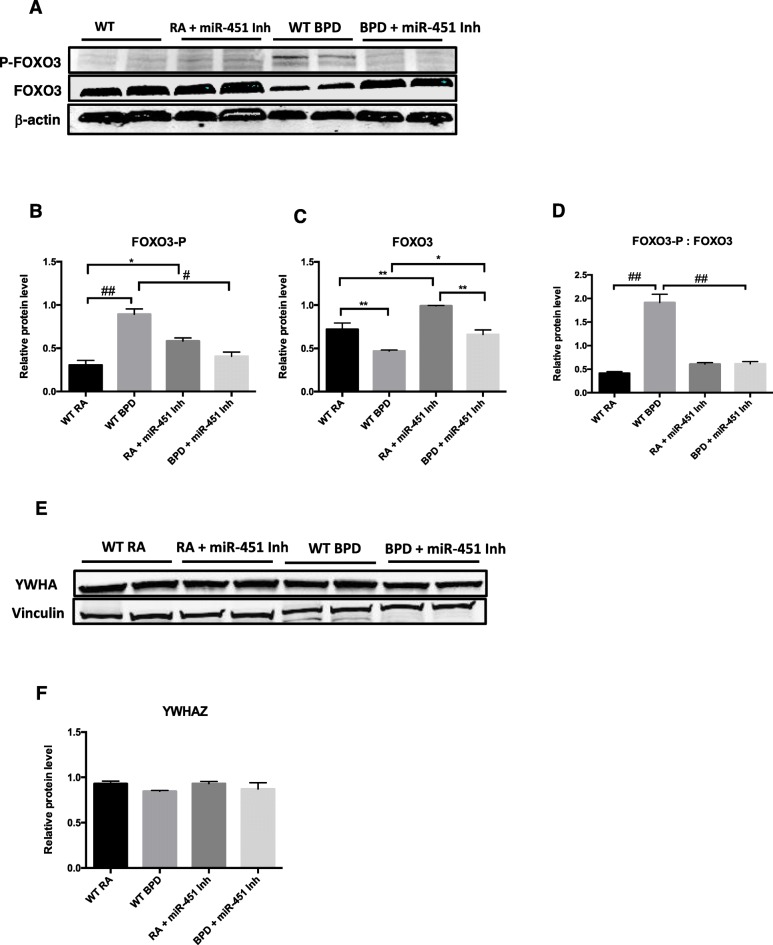
Effect of a miR-451 inhibitor on the expression of FOXO3 and YWHAZ. **a** Representative western blot showing the expression of the transcription factor forkhead box O3 (FOXO3) in both the nuclear phosphorylated form (FOXO3-P) and total (FOXO3) with β-actin serving as a control. **b** and **c** Quantification of FOXO3-P and FOXO3 expression relative to β-actin. Columns are representative of values ± SEM obtained from a minimum of 4 different animals. **d** Ratio of the normalized fraction of FOXO3-P to FOXO3, columns are representative of paired values ± SEM obtained from a minimum of 4 different animals. **e** Representative western blot showing the expression of the regulatory protein YWHAZ or 1–3-3-ζ with vinculin as a control. **f** Quantification of YWHAZ expression using densitometry with values relative to vinculin, each column is representative of the mean ± SEM of values obtained from a minimum of 4 different animals

miR-451 has been shown to be involved in regulating the production of cytokines by inhibiting the transcription of the regulatory protein YWHAZ or 1–3-3-ζ that in turn inhibits nuclear localization of the transcription factor FOXO3 [19, 20]. The phosphorylated fraction of FOXO3 (FOXO3-P), that is typically localized to the nucleus, was significantly elevated in WT BPD mice compared to WT RA mice (Fig. 8a and b). Expression of FOXO3-P was however noted to be higher in miR-451 inhibitor treated RA mice compared to WT RA mice (Fig. 8a and b). The BPD mice treated with miR-451 inhibitor had significantly lower levels of FOXO3-P, compared to WT BPD mice (Fig. 8a and b). Expression of total FOXO3 was significantly decreased in the WT BPD mice compared to WT RA mice and miR-451 inhibitor treated mice (Fig. 8a and c). Interestingly, FOXO3 expression was highest in the miR-451 inhibitor RA group. The ratio of FOXO3-P to FOXO3 was significantly elevated in the WT BPD group compared to the WT RA group and significantly decreased in the miR-451 inhibitor treated BPD mice, compared to WT BPD mice (Fig. 8a and d). No differences were noted between the miR-451 inhibitor treated RA and miR-451 inhibitor treated BPD groups (Fig. 8a and d). No significant differences in the expression of YWHAZ were noted between the four different experimental groups (Fig. 8e and f).

## Discussion

During normal lung development angiogenesis is coordinated precisely, leading to a balance in factors that promote expansion with those that promote stability of the endothelial barrier [30, 31]. Normal alveolar development has been shown to be dependent on angiogenesis [4, 32, 33] and dysregulated vascular growth is a well-established feature of the BPD phenotype [1, 4]. miR-451 is a miRNA that has been shown to inhibit the expression of pro-angiogenic and anti-apoptotic mediators such as MIF, IL-6R and YWHAZ in a variety of different malignant cell types and tumors [14, 16, 18, 34, 35]. In tumors, reduced expression of miR-451 and increased expression of MIF is associated with increased likelihood of progression [15, 36]; however, in preterm infants, increased expression of MIF is linked to a reduced risk for BPD [10, 11]. Studies published by our group utilizing murine models of neonatal hyperoxia induced lung injury (HALI) and BPD have strongly suggested that the protective effect of MIF is mediated through regulation of the Ang-Tie2 axis [12, 13] and promotion of angiogenesis through upregulation of VEGF-A [37, 38]. Studies evaluating both removal of MIF and elevation of expression to supraphysiological levels have shown that both extremes are associated with the development of a pulmonary phenotype similar to that seen in hyperoxia induced BPD [12, 13]. These findings led to the consideration of potential mechanisms that regulate MIF expression in the developing lung in both physiological and pathophysiological conditions.

The goal of this study was to evaluate miR-451 as a potential contributor to the pathogenesis of BPD and to investigate the possibility that MIF signaling pathways in the developing lung could be subject to miRNA mediated regulation. We chose to evaluate miR-451 expression using both an in-vitro model of MLECs exposed to hyperoxia and an in-vivo murine model of severe hyperoxia induced BPD. Endothelial cells were selected due to our interest in the effect of miR-451 on angiogenesis. We have demonstrated that miR-451 is upregulated in both MLECs exposed to hyperoxia and in lung tissues of NB mice exposed to 100% O_2_ during the critical saccular phase of lung development. This rise in miR-451 expression in response to hyperoxia coincides with the decrease in MIF expression previously reported by our group using the same experimental model of hyperoxia induced BPD [12] and is consistent with published findings that miR-451 is one of several miRNAs differentially regulated in a murine model of hyperoxia-induced BPD [39].

Treatment of NB mice with a miR-451 inhibitor was found to be associated with mitigation of the BPD phenotype indicated by reduced mean chord length, reduced septal thickness and increased mean radial alveolar counts relative to WT BPD mice. Treatment with a miR-451 inhibitor was also associated with a significant reduction in RVH similar to that noted in MIF overexpressing transgenic mice exposed to a model of HALI [13]. Inhibition of miR-451 was also shown to partially preserve vascular growth in BPD mice and reduce vascular remodeling indicating that the rise in miR-451 noted in MLECs and murine lung tissues may contribute to the dysregulated vascular growth that is part of the BPD phenotype.

Regulation of the balance of vascular growth factors in the developing lung is known to be critical in the creation of the extensive network of blood vessels necessary to support alveolarization [30, 31]. The Ang-Tie2 axis has been shown to play an essential role in maintaining both vascular homeostasis and expansion during lung development [31, 40]. As previous work published by our group has indicated that the known miR-451 target MIF acts as a regulator of Ang signaling, we proceeded to evaluate the effect of a miR-451 inhibitor on the expression of MIF, Ang1, Ang2 and the Ang receptor Tie2. MIF expression did not decrease in MLECs exposed to hyperoxia; however, administration of a miR-451 inhibitor was associated with increased expression of MIF. This finding differs from results shown in murine BPD lung specimens where MIF decreases in response to hyperoxia and expression is preserved following treatment with a miR-451 inhibitor. Discrepancies in the expression of both Ang1, Ang2 and the Ang receptor, Tie2 were also noted between in-vitro and in-vivo models. Ang1 acts via the Tie2 receptor to increase expression of vascular adhesion molecules, inhibit apoptosis and promote stability of the endothelial cell barrier [31, 41]. Ang2 competes with Ang1 for Tie2 binding sites and has the opposite effect; loosening connections between endothelial cells in order to promote branching [31, 40]. Although no changes in Ang1 expression were noted in MLECs exposed to hyperoxia, Ang1 expression was noted to be significantly decreased in WT BPD mice relative to the WT RA group. This decrease in Ang1 expression is consistent with the pattern noted in both experimental models of BPD [12] and in preterm infants who go on to have adverse pulmonary outcomes [42, 43]. Administration of a miR-451 inhibitor to NB mice was associated with a significant increase in Ang1 expression that was partially maintained following exposure to hyperoxia. No change was noted in the expression of Ang1 in MLECs either following exposure to hyperoxia or to a miR-451 inhibitor. Ang2 expression was found to increase in both MLECs exposed to hyperoxia and in the lungs of BPD mice. This result is consistent with previous reports in the literature that identify Ang2 as a pathogenic regulator in the response to hyperoxia [29, 44] and a potential biomarker denoting increased risk for BPD [45, 46]. miR-451 inhibitor administration was associated with a decrease in Ang2 in MLECs exposed to hyperoxia; however, Ang2 expression was significantly increased in the lungs of both RA and BPD mice who were treated with the antagomir. Administration of the miR-451 inhibitor was also associated with increased expression of the Ang receptor Tie2 in murine lung tissues. No changes in Tie2 expression noted in MLECs. Previous work has demonstrated that pulmonary vascular development is driven by interactions between alveolar epithelial and endothelial cells [47] and it is possible that the different expression patterns noted in the in-vivo model could have occurred due to the influence of other pulmonary cell types. MIF is constitutively expressed in nearly every cell type [8] and it is also possible that other pulmonary cell types are making a greater contribution to MIF expression during normoxic conditions. Interactions between different cell types occurring in-vivo could also explain the differences noted in the expression of the Ang proteins and their receptor Tie2.

miR-451 has been shown to inhibit angiogenesis through targeting MIF, calcium-binding protein 39 (CAB39) and IL-6R mediated pathways that influence expression of VEGF-A [16, 35, 48]. In our study, administration of a miR-451 inhibitor was also associated with increased expression of VEGF-A. VEGF expression has been shown to be decreased in both experimental models of BPD and in preterm infants who develop adverse pulmonary outcomes [32, 49–51] and gene therapy with VEGF has been shown to improve alveolarization in a rat model of hyperoxic lung injury [32]. The pro-apoptotic activity of Ang2 is also known to be reduced in the presence of increased levels of VEGF [52] which may explain in part why treatment with a miR-451 was associated with both increased expression of Ang2 and mitigation of the BPD phenotype. The increase in Ang1 expression associated with administration of a miR-451 inhibitor could also potentially balance the deleterious effects of Ang2. The Ang1:Ang2 ratio which is known to decreased in the presence of hyperoxia [12, 25] was significantly improved in the lungs of BPD mice treated with a miR-451 inhibitor. Increased expression of Tie2 noted following miR-451 inhibitor administration might also contribute to the relative improvements in vascular growth and alveolarization seen in the BPD group.

BPD associated PAH is characterized by various forms of vascular remodeling, and elastin being a major component of blood vessels is easily visualized using Verhoeff’s stain. In the present study, we found that the arterial walls were thickened and dense with abundant deposition of collagen following exposure to hyperoxia; however, treatment with the miR-451 inhibitor was associated with a significant decrease in arterial wall thickness and a reduction in the deposition of collagen. These findings indicate remodeling of the large vessels towards a state of normalcy in the presence of a miR-451 inhibitor. Taken together with the significant reduction in RVH and relative preservation of in vascular density in BPD mice treated with the miR-451 inhibitor these findings suggest that increased expression of miR-451 in the presence of hyperoxia may contribute to the dysregulation of vascularization that is part of the BPD pulmonary phenotype.

In addition to anti-angiogenic and pro-apoptotic activities, miR-451 has also been shown to downregulate the inflammatory responses in a variety of different experimental models [20, 53, 54]. In our model of hyperoxia induced BPD, treatment with a miR-451 inhibitor was also associated with several indications of an increased inflammatory response including elevation of BAL total neutrophil count, elevation in BAL total protein and increased expression of pro-inflammatory cytokines IL-6 and IL-1β. MIF has been shown to promote arrest and transmigration of neutrophils across the endothelial cell barrier through interactions with the chemokine receptors CXCR2 and CXCR4 [55, 56]. Therefore, upregulation of MIF expression in the presence of a miR-451 inhibitor could account for the increased numbers of neutrophils present in the BAL specimens. miR-451 has also been shown to reduce production of pro-inflammatory cytokines in murine dendritic cells exposed to influenza virus by inhibiting the expression of the regulatory protein YWHAZ [20]. YWHAZ acts as an inhibitor of the zinc finger protein ZFP36 and the inhibitory transcription factor FOXO3. Inhibition of miR-451 in this model was associated with increased expression of YWHAZ, decreased nuclear expression of FOXO3 and increased production of IL-6, among other cytokines [20]. In the current study we did note that IL-6 and IL-1β expression was not only significantly increased in the WT BPD group but also in both groups of miR-451 inhibitor treated mice. We did note changes in the expression of FOXO3 with significantly reduced expression of the phosphorylated nuclear form in the miR-451 inhibitor treated BPD mice when compared to the WT BPD mice. This appears to be occurring independently of YWHAZ. The inferences we can draw from these findings are limited as western blotting was performed on whole lung tissue lysates rather than discrete nuclear and cytoplasmic fractions; however, regulation of FOXO3 by miR-451 may be an area for future investigation in this BPD model.

An excessive inflammatory response has been established as an important contributor to the pathogenesis of BPD [57, 58]; however, the impact of pro-inflammatory cytokines on the NB lung has been shown to be dependent on the timing and duration of exposure [59, 60]. IL-6 in addition to having pro-inflammatory activities, also plays an important role in anti-inflammatory and regenerative processes [61]. When NB IL-6 transgenic mice were studied in a model of hyperoxia-induced lung injury, absence of IL-6 was found to be associated with higher levels of pro-inflammatory cytokines and increased apoptosis in comparison to WT controls [62]. IL-6 is also known to act via hypoxia-inducible factor (HIF)-1α to increase VEGF expression [63] and this process has been shown to be inhibited by miR-451 mediated regulation of IL-6R expression in colorectal cancer cells [16]. It is therefore possible to explain why disruption of miR-451 mediated inhibition of both inflammation and angiogenesis could result in relative improvements in lung morphometry and vascular growth in murine lungs exposed to hyperoxia.

Our study does have several limitations. Although the in-vivo model we used correlates with the pathological phenotype and long term consequences of neonatal lung disease [22, 59, 64] these experiments cannot fully replicate the combination of factors that contribute to the development of BPD. The decision to randomize animals from the same litter to the same experimental group is another limitation of our study. As the sample size of the mice dams used for these experiments was low, we cannot exclude the possibility of the influence of maternal genetic variation on the differences we noted. As miR-451 acts on multiple targets, at this stage we are not yet able to ascribe the benefits of miR-451 inhibition exclusively to upregulation of a specific target or pathway. Although comparison of our results with those obtained from the study of MIF over-expressing transgenic mice (MIFTG) [12, 13] lead us to speculate that MIF is involved, a relationship between miR-451 and MIF within the context of BPD can only be suggested, as modulation of this signaling pathway by other molecules is probably playing a role. Although the relative improvements seen in alveolarization and angiogenesis are impressive, more work needs to be performed to define the specific pathways that contribute to these findings.

## Conclusions

In summary, we have demonstrated for the first time that miR-451 is upregulated in the presence of hyperoxia in both MLECs and in the lung tissues of mice studied using a model of hyperoxia-induced BPD. Inhibition of miR-451 expression resulted in improvement in the lung architecture, a reduction in RVH, mitigation of pulmonary vascular wall thickening and relative preservation of pulmonary vascular growth. Inhibition of miR-451 was associated with preserved expression of MIF in the BPD group and increased expression of VEGF-A, Ang1, Ang2 and the Ang receptor, Tie2. Further investigation of the role of miR-451 in the pathogenesis of BPD has the potential to enhance our understanding of mechanisms regulating MIF signaling in the developing lung and may identify novel therapeutic targets.

## Supplementary information

**Additional file 1: Figure S1.** miR-451 expression is upregulated in fetal MLECs following exposure to hyperoxia. RNA was extracted from fetal MLECs grown in room air and exposed to hyperoxia (60% O_2_) for 16 h. miR-451 expression was evaluated using RT-qPCR. *N* = 3, in each group. Fetal MLECs: fetal mouse lung endothelial cells, RA: room air, Hyp-16H: hyperoxia with 60% O_2_ for 16 h; RA: room air; * *p* < 0.05 Data are expressed as mean ± SEM.

**Additional file 2: Figure S2.** Effect of hyperoxia on expression of miR-451 in MLE12 cells. RNA was extracted from MLE12 cells grown in room air and exposed to hyperoxia (95% O_2_) for either 4 or 16 h. miR-451 expression was evaluated using RT-qPCR. N = 3, in each group. MLE12 cells: mouse lung epithelial cells, RA: room air, Hyp – 4H: hyperoxia for 4 h; Hyp-16H: hyperoxia for 16 h; *p* = 0.13. Data are expressed as mean ± SEM.

## Data Availability

The datasets used and/or analyzed during the current study are available from the corresponding author on reasonable request.

## Abbreviations

Ang1: angiopoietin 1
Ang2: angiopoietin 2
BAL: bronchoalveolar lavage
BPD: bronchopulmonary dysplasia
CAB39: calcium-associated binding protein 39
DAPI: 4′,6-diamidino-2-phenylindole
FOXO3: forkhead box protein O3
FOXO3-P: phosphorylated fraction of forkhead box protein O3
HALI: hyperoxia induced lung injury
H&E: hematoxylin and eosin
HIF-1α: hypoxia inducible factor-1 alpha
HPF: high powered field
Hyp: hyperoxia
IL: interleukin
IL-6R: interleukin-6 receptor
IVS: interventricular septum
LV: left ventricle
MIF: macrophage migration inhibitory factor
MIFKO: MIF null mutant mouse
MIFTG: transgenic mouse with inducible overexpression of MIF
miR-451: microRNA-451
miR-451 Inh: miR-451 inhibitor
MLEC: Murine lung endothelial cells
MLE12: Murine lung epithelial cells
NB: newborn
PAH: pulmonary arterial hypertension
PN: post-natal day
RA: room air
RDS: respiratory distress syndrome
RV: right ventricle
RVH: right ventricular hypertrophy
SEM: standard error of the mean
Tie2: tyrosine kinase with Ig and epidermal growth factor homology domains receptor 2
VEGF-A: vascular endothelial growth factor
vWF: Von Willebrand factor
WT: wild type
YWHAZ: Tyrosine 3-Monooxygenase/Tryptophan 5-Monooxygenase Activation Protein Zeta
