# Correction to: Inhibition of microRNA-451 is associated with increased expression of Macrophage Migration Inhibitory Factor and mitigation of the cardio-pulmonary phenotype in a murine model of Bronchopulmonary Dysplasia

**DOI:** 10.1186/s12931-020-01378-0

**Published:** 2020-05-23

**Authors:** Margaret Gilfillan, Pragnya Das, Dilip Shah, Mohammad Afaque Alam, Vineet Bhandari

**Affiliations:** 1 Department of Pediatrics, Drexel University College of Medicine, Philadelphia, PA 19103 USA; 2 St Christopher’s Hospital for Children, Philadelphia, PA 19134 USA; 3 Neonatology Research Laboratory, Education and Research Building, Cooper University Hospital, (Room #206), Camden, NJ 08103 USA; 4 Temple University, Philadelphia, PA 19140 USA; 5 Pediatrics, Obstetrics and Gynecology and Biomedical Sciences, Cooper Medical School of Rowan University, Camden, NJ 08103 USA; 6 Neonatology, The Children’s Regional Hospital at Cooper, One Cooper Plaza, Camden, NJ 08103 USA

**Correction to: Respir Res (2020) 21:92**

**https://doi.org/10.1186/s12931-020-01353-9**

Following publication of the original article [1], the authors noted that the measurement units on the y-axes on Figs. 4B, C and 6E had been incorrectly published as “mm”, instead of “µm”. The original article has been corrected.

**Figure 1:**
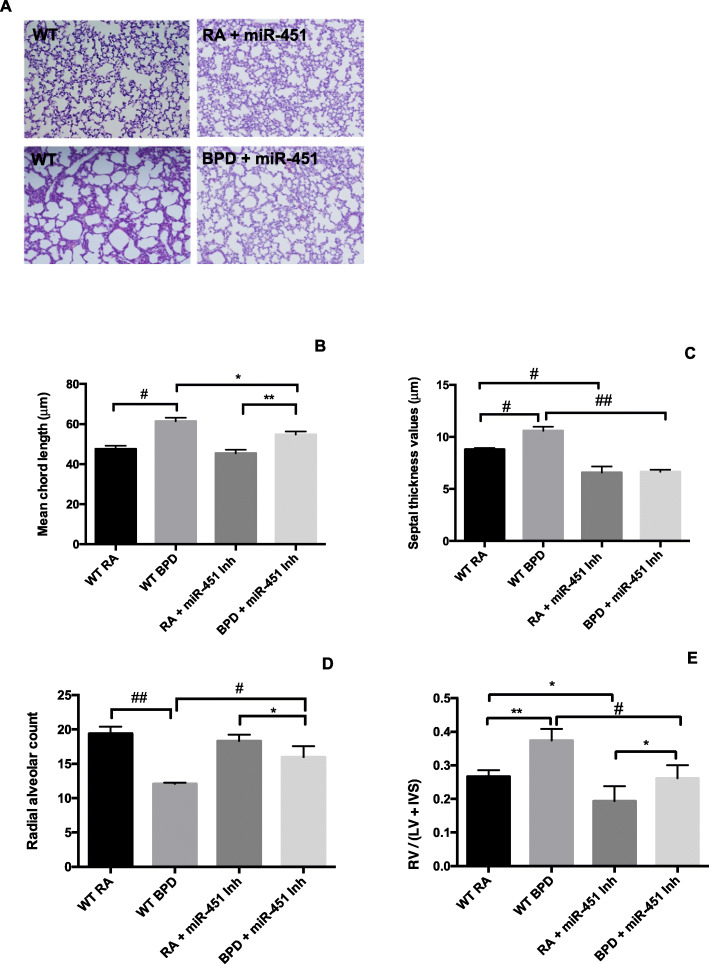
Fig. 4

**Figure 2:**
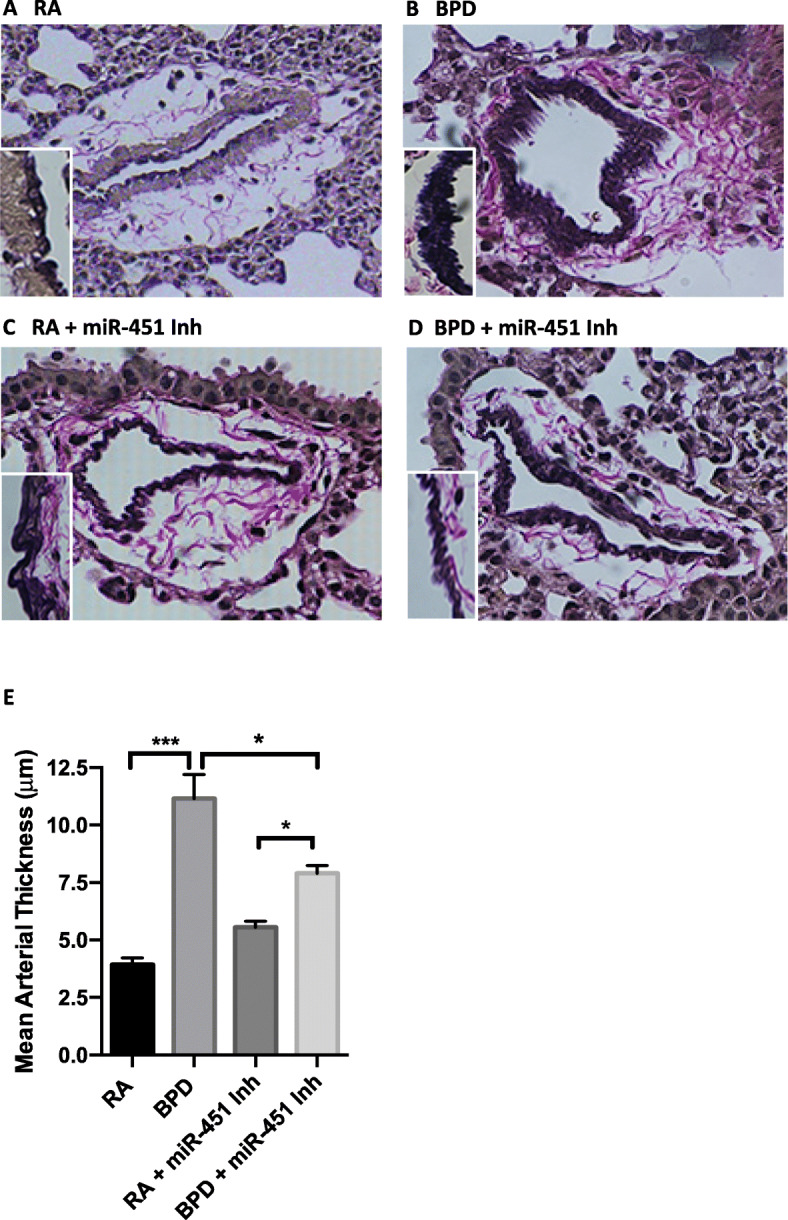
Fig. 6
