# Supplementary material for: Inhibition of microRNA-451 is associated with increased expression of Macrophage Migration Inhibitory Factor and mitgation of the cardio-pulmonary phenotype in a murine model of Bronchopulmonary Dysplasia
Source: Respir Res. 2020 Apr 22;21:92. doi: 10.1186/s12931-020-01353-9 (PMC7178994; doi:10.1186/s12931-020-01353-9)
Supplement: Supplementary file 1 — Additional file 1: Figure S1. miR-451 expression is upregulated in fetal MLECs following exposure to hyperoxia. RNA was extracted from fetal MLECs grown in room air and exposed to hyperoxia (60% O2) for 16 h. miR-451 expression was evaluated using RT-qPCR. N = 3, in each group. Fetal MLECs: fetal mouse lung endothelial cells, RA: room air, Hyp-16H: hyperoxia with 60% O2 for 16 h; RA: room air; * p < 0.05 Data are expressed as mean ± SEM. [file 12931_2020_1353_MOESM1_ESM.docx]

**Figure 1.**

**A**

**B**
