# Supplementary figures and images for: Inhibition of microRNA-451 is associated with increased expression of Macrophage Migration Inhibitory Factor and mitgation of the cardio-pulmonary phenotype in a murine model of Bronchopulmonary Dysplasia
Source: Respir Res. 2020 Apr 22;21:92. doi: 10.1186/s12931-020-01353-9 (PMC7178994; doi:10.1186/s12931-020-01353-9)

**Figure 2.**

**RA + miR-451 Inh Hyperoxia Hyp + miR-451 Inh**

**RA**


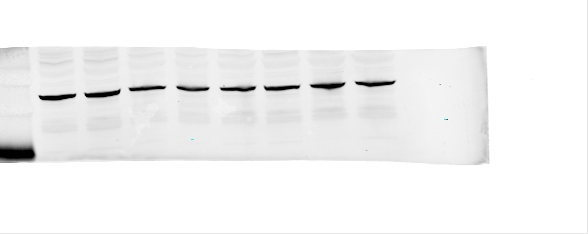


**β-Actin**


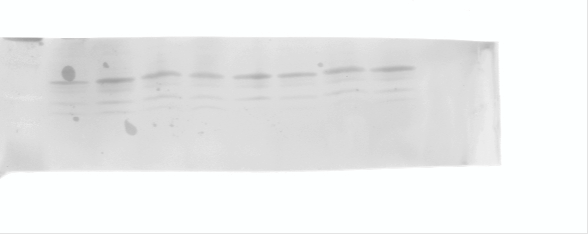


**MIF**


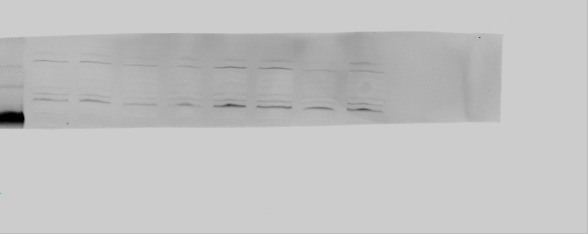


**Ang1**


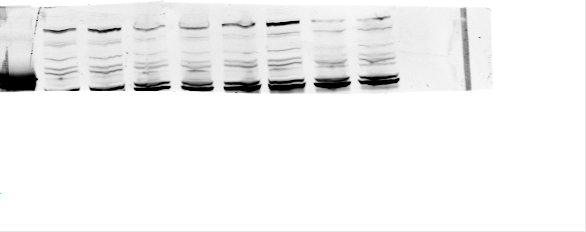


**Ang2**

**TIE 2**


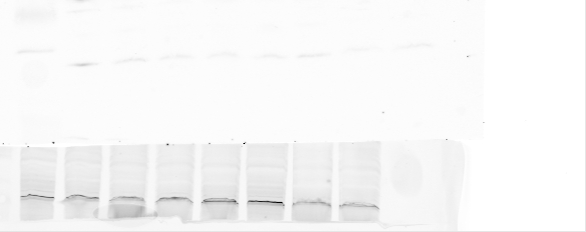


**E**

**D**

**B**

**C**

**C**

**C**

**F**

**B**

Supplement: Supplementary file 2 — Additional file 2: Figure S2. Effect of hyperoxia on expression of miR-451 in MLE12 cells. RNA was extracted from MLE12 cells grown in room air and exposed to hyperoxia (95% O2) for either 4 or 16 h. miR-451 expression was evaluated using RT-qPCR. N = 3, in each group. MLE12 cells: mouse lung epithelial cells, RA: room air, Hyp – 4H: hyperoxia for 4 h; Hyp-16H: hyperoxia for 16 h; p = 0.13. Data are expressed as mean ± SEM. [file 12931_2020_1353_MOESM2_ESM.docx]
